# A Scalable Method to Fabricate 2D Hydrogel Substrates for Mechanobiology Studies with Independent Tuning of Adhesiveness and Stiffness

**DOI:** 10.3390/mps7050075

**Published:** 2024-09-26

**Authors:** Alessandro Gandin, Veronica Torresan, Tito Panciera, Giovanna Brusatin

**Affiliations:** 1 Department of Industrial Engineering, University of Padova, Via Marzolo 9, 35131 Padova, PD, Italy; alessandro.gandin@unipd.it (A.G.); veronica.torresan@unipd.it (V.T.); 2 Consorzio INSTM, Padova RU, Via Marzolo 9, 35131 Padova, PD, Italy; 3 Department of Molecular Medicine, University of Padova, Via Ugo Bassi 58/B, 35131 Padova, PD, Italy; tito.panciera@unipd.it

**Keywords:** PEG-hydrogels, 2D substrates, stiffness, adhesiveness, mechanobiology

## Abstract

Mechanical signals from the extracellular matrix are crucial in guiding cellular behavior. Two-dimensional hydrogel substrates for cell cultures serve as exceptional tools for mechanobiology studies because they mimic the biomechanical and adhesive characteristics of natural environments. However, the interdisciplinary knowledge required to synthetize and manipulate these biomaterials typically restricts their widespread use in biological laboratories, which may not have the material science expertise or specialized instrumentation. To address this, we propose a scalable method that requires minimal setup to produce 2D hydrogel substrates with independent modulation of the rigidity and adhesiveness within the range typical of natural tissues. In this method, norbornene-terminated 8-arm polyethylene glycol is stoichiometrically functionalized with RGD peptides and crosslinked with a di-cysteine terminated peptide via a thiol–ene click reaction. Since the synthesis process significantly influences the final properties of the hydrogels, we provide a detailed description of the chemical procedure to ensure reproducibility and high throughput results. We demonstrate examples of cell mechanosignaling by monitoring the activation state of the mechanoeffector proteins YAP/TAZ. This method effectively dissects the influence of biophysical and adhesive cues on cell behavior. We believe that our procedure will be easily adopted by other cell biology laboratories, improving its accessibility and practical application.

## 1. Introduction 

Longstanding oversights regarding the relationship between cell behavior and the mechanics of their biological environment have been largely due to the use of rigid, unphysiological culturing substrates made of solid plastic. 

On these substrates, cells display aberrant behavior characterized by distorted phenotypes with abnormal polarization, excessive proliferation, and unconstrained spreading, skewing natural cellular responses to their environment.

This distortion in cellular perception of surrounding mechanics began to shift with advancements in biomaterials science. Notably, the development of hydrogels—water-swollen polymers that closely mimic the extracellular matrix (ECM)—has been transformative in cellular biology, particularly in mechanobiology [1,2,3,4]. This shift marks a significant paradigm change, aligning experimental conditions more closely with physiological realities.

Hydrogels have emerged as pivotal tools in cell biology, with recent publications detailing their development and application [5,6,7,8,9,10]. Notably, 2D hydrogel substrates specifically engineered for cell culture [3,11,12,13,14] have demonstrated that substrate stiffness and adhesiveness crucially influence cell behavior and gene expression. 

However, studies aiming to dissect the separate impacts of these two parameters are scant and often yield incomparable results due to variations in substrate composition and synthesis techniques [3,5,10,15,16,17,18,19,20,21]. Despite these promising developments, the full potential of these advanced materials remains untapped, primarily because of the absence of standardized, easily reproducible hydrogel synthesis protocols that can be adopted by any biological laboratory.

Tse and Engler [14] significantly advanced the field by introducing an excellent protocol to create polyacrylamide hydrogels. This protocol has become a fundamental guide for synthesizing mechanically tunable substrates essential for cell biology studies. Today, the vast majority of studies on substrate mechanics are limited to replicating the use of PAA-based substrates, with only minimal variations. 

Beyond polyacrylamide, polyethylene glycol (PEG) has also emerged as a versatile biomaterial due to its range in molecular weights, structures, functionalities, and chemistries, notably including “click” chemistry. This adaptability has spurred the development of synthesis methods based on a LEGO-like approach, where modular “building blocks” are assembled using biorthogonal and biocompatible chemistries to craft precise and reproducible hydrogel networks and functionalities. Despite these innovations, comprehensive and detailed protocols for controlled and standardized synthesis of PEG hydrogels remain scarce, which hampers their broader adoption in cell biology. Additionally, existing methods typically produce gels with limited stiffness ranges, either overly soft (<1 kPa) or excessively stiff (>4–5 kPa).

To address this gap, we introduce a protocol for synthesizing PEG-based hydrogels, designed for straightforward implementation with equipment commonly available in biomaterials and biological laboratories. These hydrogels serve as versatile substrates for cell culture and mechanobiology studies, offering independent control over mechanical and adhesive properties. In particular, we use norbornene-terminated 8-arm polyethylene glycol, which is click-reacted with RGD cell adhesive motif and di-cysteine terminated peptides, via a norbornene–cystein thiol–ene reaction. This synthesis approach allows precise and independent modulation of gel substrate rigidity and adhesivity, by adjusting the crosslinking of the PEG macromer and the density of integrin-binding sites through stoichiometric control of adhesive peptides. Gel stiffness can be tuned from 0.3 to 14 kPa, as outlined in our previous publication [22].

These characteristics facilitate distinct analysis of how stiffness and adhesivity independently influence cell behavior.

## 2. Experimental Design

Here we present a protocol for the synthesis of polyethylene glycol hydrogels based on multi-arm PEG-norbonene terminated bifunctional peptides as crosslinkers and monofunctional peptides to tune cell adhesiveness. 

**Figure 1:**
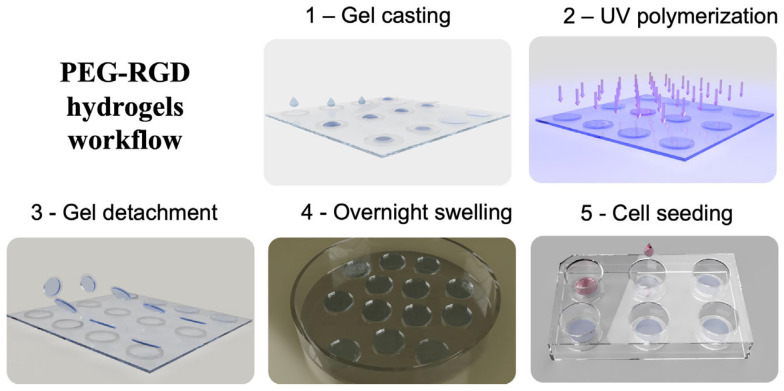
Schematic protocol workflow for hydrogel synthesis. Taken from [22] with minor modification.

The protocol describes the procedure to obtain 2D flat hydrogels for cell culture with tunable stiffness and integrin binding domains, detailing the following steps: chemical functionalization for non-adhesive glass substrate, chemical functionalization for gel-binding glass coverslips, polymerization setup preparation, hydrogel synthesis by a light-mediated procedure, and hydrogel sterilization for cell culture (Figure 1).

This protocol will be useful for biologists and materials scientists interested in the investigation of the cell-matrix interactions and biological mechanisms at the root of cellular mechanobiology on physiological ECM mimicking substrates.

A table reporting the time required to complete each step is provided here for 10 gels (Table 1). Time estimations for the synthesis of 30 and 50 gels is reported in the expected results section.

### 2.1. Materials

- PlusOne Repel-silane ES (VWR Avantor, Milano, Italy, product number: 17-1332-01)
- Deionized water
- 3-(Trimethoxysilyl)propyl methacrylate (Merck KGaA, Darmstadt, Germany, catalog number: 440159)
- Sodium hydroxide (Sigma-Aldrich, catalog number: 221465)
- MilliQ ultrapure water
- PBS 1X -/-
- Ethanol (Sigma-Aldrich, catalog number: 32221-1L-M)
- Acetic acid glacial (Sigma-Aldrich, catalog number: 695092)
- Acetone (Sigma-Aldrich, catalog number: 179124)
- 8-Arm PEG-Norbornene, MW 40k (CreativePEGWorks, Chapel Hill, NC, United States, catalog number: PSB-8310)
- Synthetic crosslinking peptide. Sequence: CRDGQPGYSGQDRC (CRIBI Biotechnology Center, Padova, Italy)
- Synthetic cell-adhesive peptide. Sequence: GRGDSPC (CRIBI Biotechnology Center)
- Synthetic non-adhesive peptide. Sequence: GRDGSPC (CRIBI Biotechnology Center)
- Lithium phenyl-2,4,6-trimethylbenzoylphosphinate (Sigma-Aldrich, catalog number: 900889)

### 2.2. Equipment

- Hotplate (IKA-Werke GmbH & Co. KG, Staufen, Germany, RCT Basic)
- Plasma cleaner (Harrick Plasma, Ithaca, NY, United States, PDC-002-CE)
- Sharp-tipped tweezers
- Laminar flow cell culture hood
- UV curing lamp (DELO, Windach, Germany, Delolux 20)

### 2.3. Laboratory Supplies

- ∅18 mm round glass coverslips (Avantor VWR, Milano, Italy, catalog number: 631-1580)
- Rectangular glass slides (VWR Avantor, Milano, Italy, catalog number: 631-9460)
- PDMS sheeting 0.010″ (Specialty Manufacturing Inc., Saginaw, MI, United States, catalog number: 70P001-200-010)
- Low retention tips (Axygen™, Corning, Corning, NY, United States, CATALOG NUMBER: TR-222-C-L-R-S)

## 3. Procedure

### 3.1. Dimethyl Silane Functionalization (Non-Adhesive)—Time Needed: 230 Min

**Figure 2:**
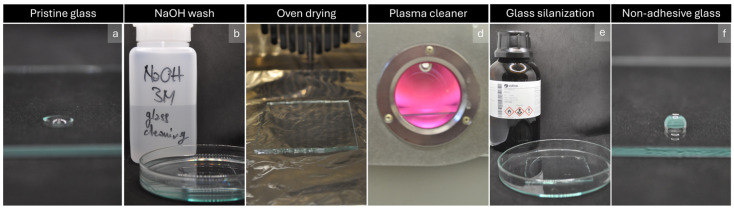
(**a**) hydrophilic glass slide; (**b**) wash with NaOH 3 M; (**c**) dehydration of the substrate; (**d**) activation of the surface with plasma cleaner; (**e**) substrate silanization using Repel-silane; (**f**) hydrophobic glass substrate.

1. Wash, submerging the glass slide, with 3 M NaOH for 20′ (Figure 2b).
2. Collect NaOH solution in a proper container. This solution can be reused for future glass cleaning.
3. Rinse, submerging the glass slide in deionized water two or three times until the pH of the washing solution is 6–7.
4. Put the glass slides on a hotplate at 150 °C to dehydrate the substrates for 30′ (Figure 2c).
5. Let the clean glasses cool down to room temperature.
6. Treat the surface with plasma cleaner for 90″ to activate the substrates (Figure 2d).
7. Cover the surface with Repel-silane (Figure 2e).
8. Let the functionalization proceed for 15′; add solution if necessary to keep the surface wet.
9. Rinse the glass slides with ethanol.
10. Put the washed slides on a hotplate or in an oven at 120 °C for 30′.
11. **PAUSE STEP:** Functionalized glass can be stored in a dry and clean box for two weeks before use.

### 3.2. Methacrylate Silane Functionalization (Adhesive)—Time Needed: 90 Min

12. Wash, submerging the coverslips, with 3 M NaOH for 20′.
13. Collect NaOH solution in a proper container. This solution can be reused for future glass cleaning.
14. Rinse, submerging the glass coverslips in deionized water two or three times until the pH of the washing solution is 6–7.
15. Put the coverslips on a hotplate at 150 °C to dehydrate the substrates for 10′.
16. Let the clean glasses cool down to room temperature.
17. Treat the surface with plasma cleaner for 90″ to activate the substrates.
18. Put a small drop (e.g., 20 μL) of TMSPM solution on each coverslip, covering the surface.
19. Let the functionalization proceed for 15′; add solution if necessary to keep the surface wet.
20. Rinse the coverslips three times with acetone.
21. Put the washed coverslips on a clean support and let them air dry.

### 3.3. Polymerization Set-Up Preparation—Time Needed: 30 Min

22. Cut circular rings from the silicone sheet and wash them with pure ethanol.
23. Place them inside a plastic petri dish filled with pure ethanol until ready to use.
24. Arrange the rings on the functionalized side of the glass slides. Keep a distance between the rings of about 5 mm.
25. Let the ethanol evaporate to assure a tight adhesion.

### 3.4. Hydrogel Fabrication—Time Needed: 30 Min

26. Prepare a stock solution of 8-arm Norbornene-terminated PEG in 1X PBS to a final concentration of 250 mg/mL.

Note: PEG solution has high viscosity. To avoid the withdrawal of the wrong volume, use the low-retention tips.

27. Prepare a stock solution of LAP initiator in 1X PBS to a final concentration of 31.7 mg/mL.
28. Prepare a stock solution of the crosslinker peptide in MilliQ water to a final concentration of 40 mg/mL.
29. Prepare a stock solution of the adhesive and non-adhesive peptide in MilliQ water to a final concentration of 37.5 mg/mL.

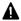
 **CRITICAL STEP:** Resuspend the cysteine-terminated peptides right before the hydrogel synthesis. A prolonged period in water can lead to oxidation of the terminal cysteines, leading to defective crosslinking and RGD functionalization of the hydrogel.

Note: residual peptide solutions should be discarded after gel fabrication. Prolonged storage at room temperature or −20 °C can lead to cysteine oxidation.

30. Mix stock solutions and 1X PBS following the order reported in Table 2 to obtain the desired elastic moduli.

31. **OPTIONAL STEP:** Adhesive peptide can be substituted totally or partially using a stock solution with the same concentration of non-adhesive peptide to tune the adhesiveness of the gel.

Note: It is important to keep the total concentration of adhesive peptide (RGD) and scramble peptide (RDG) fixed, to not alter the mechanical properties of the gels as reported in Table 2. Decreasing or increasing the total amount of monofunctional peptides can lead to higher or lower moduli, respectively, due to changes in the crosslinking degree. Excessive RGD or RDG could lead to ineffective crosslinking hindering the formation of the hydrogel. The use of RDG only, or the absence of peptide functionalization, does not allow cell attachment, making further analysis impossible.

**Figure 3:**
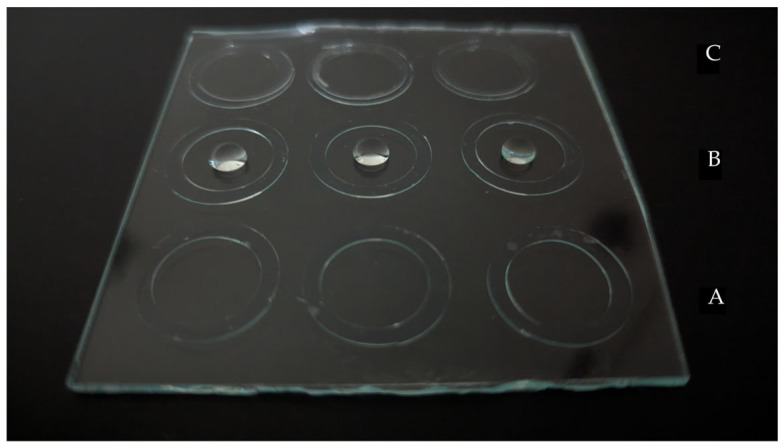
Photo of the set-up of gel casting step. Line A, shows the gasket attached on the non-adhesive glass. Line B shows the deposition of the prepolymer solution inside the gasket and line C the final setup with the adhesive glass on top of the prepolymer solution.

32. Place a 60 μL drop inside each PDMS ring and place a silanized glass coverslip on top, with the adhesive side pointing toward the prepolymer solution, as shown in Figure 3.

33. Expose the prepolymer solution to 400 nm collimated UV light, following intensity and exposure time reported in Table 3.

34. Using sharp-tipped tweezers or a scalpel, gently lift the upper coverslip, detaching the gel from the lower substrate.
35. Place the gels in a petri dish filled with 1X PBS and leave them at room temperature overnight to reach the swelling equilibrium.
36. The following day, wash the gels with 1X PBS to get rid of all the unpolymerized molecules and residual initiator.

Note 1: For the preparation of different compositions, it is recommended to combine in the same polymerization step the compositions that require the same synthesis time, starting with the stiffer or the faster ones. 

Note 2: The thickness of hydrogel before the overnight swelling is 250 μm. Changing the thickness of the hydrogel may require slightly different UV light exposure times. 

### 3.5. Hydrogel Sterilization—Time Needed: 20 Min (for 10 Hydrogels)


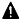
 **CRITICAL STEP:** From this point on, work under a laminar flow sterile hood.

37. Prepare a small sponge soaked with a 70% ethanol solution (see recipe 3) inside a petri dish.
38. Take the gels one by one and clean the bottom of the glass substrate by tapping gently on the sponge.
39. Place the gel inside a 12-well multiwell.
40. Repeat steps 35 and 36 for all the hydrogels.
41. Fill the wells with 1 mL of sterile 1X PBS.
42. Sterilize the gels with the multiwell lid open under the UV lamp of the cell culture hood for 15 to 20 min.
43. Gels can be maintained in 1X PBS until cell seeding.
44.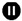 **PAUSE STEP:** Sterilized hydrogels can be kept up to two days in 1X PBS before seeding. However, it is recommended to prepare hydrogels fresh for every experiment and use them the day after the synthesis to reduce the risk of contaminations and unwanted changes in mechanical properties due to hydrolysis and depolymerization.

## 4. Expected Results

### 4.1. Hydrogel Outcome and Physical Characteristics

**Figure 4:**
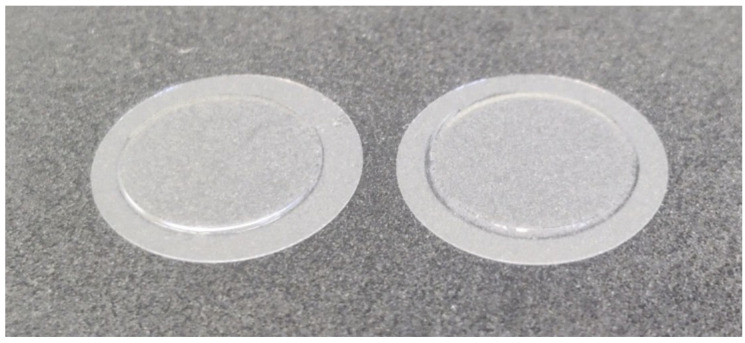
Example of synthetized hydrogels. Round glass coverslips are used as a rigid, transparent substrate to anchor the polymerized hydrogel.

An example of the hydrogels obtained from following this protocol is reported in Figure 4. The expected outcome is round flat hydrogels with a homogeneous and defined area controlled by the dimension of the PDMS gasket. Gels should be strongly coupled with the glass substrates, which helps the handling of the gels during cell seeding and the following downstream biological assays.

Thickness of both glass coverslips and hydrogels can be optimized according to the type of analysis. Thicker gels with #1 glass coverslips are easier to handle and can be used to perform immunofluorescence staining. Thin (thickness #0) glass coverslips and thinner PDMS gaskets could potentially be used to prepare hydrogels for high-resolution live imaging.

**Figure 5:**
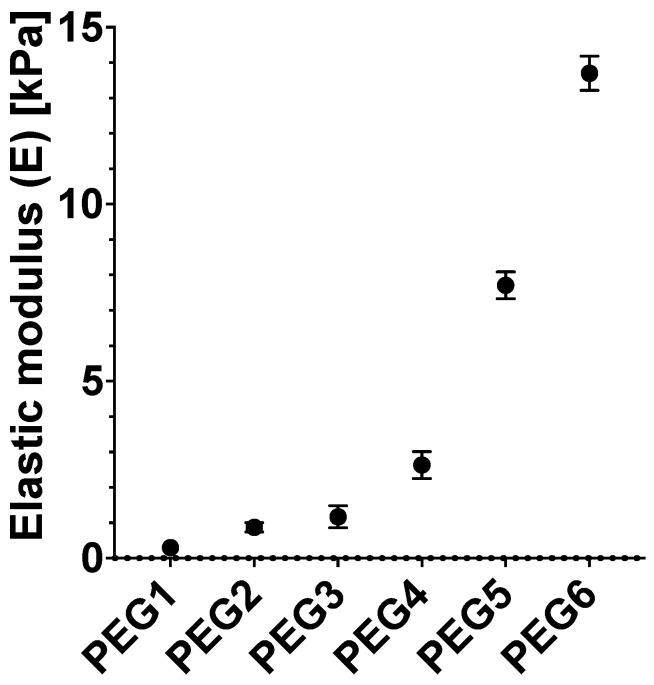
Elastic moduli assessed through micropipette aspiration. Experimental methods for mechanical analysis are described in a previous publication [23]. Values are reported as means. Error bars represent the standard deviation.

Figure 5 shows the mechanical properties (elastic modulus) assessed with the micropipette aspiration techniques, following the method reported in Gandin et al. [23]. To carry out the analysis, the thickness of the gel was set to 3 mm by adapting the PDMS thickness. Mechanical properties of PEG hydrogels can be evaluated with other methods such as uniaxial compression, atomic force microscopy, and microindentation. Sample geometry should be tailored accordingly to the requirements of the testing methods.

**Figure 6:**
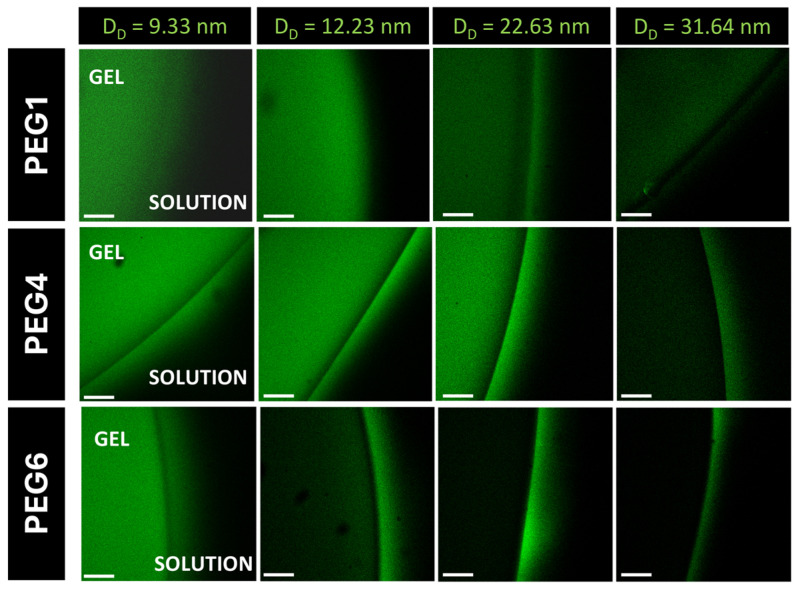
Mesh sizes of three different gel compositions evaluated through confocal imaging analyzing the diffusion of fluorescent dextrans with known hydrodynamic diameters (D_D_) inside the network of the gels. Scale bar 200 μm.

Moreover, the cut-off of mesh size can be evaluated by observing the exclusion of fluorescent probes of known size in a 1X PBS solution (Figure 6). Hydrogel samples were placed in a glass-bottomed dish and swollen for 24 h in 1X PBS. Then, the PBS was replaced with a solution of fluorescent dextrans with a different molecular weight, and a confocal image of the gel–solution interface was collected. To allow for probe diffusion in the material, the gel was left in the fluorescent solution for an additional 24 h. Then, after the replacement of the solution with fresh PBS, a second image was acquired to assess whether the fluorescent probes were diffused inside the material.

Finally, a more comprehensive estimation of the time to perform the synthesis is reported in Table 4. Each step of the protocol is indicated for the synthesis of 10, 30, or 50 hydrogels. 

### 4.2. Mechanobiology Application of PEG Hydrogels

**Figure 7:**
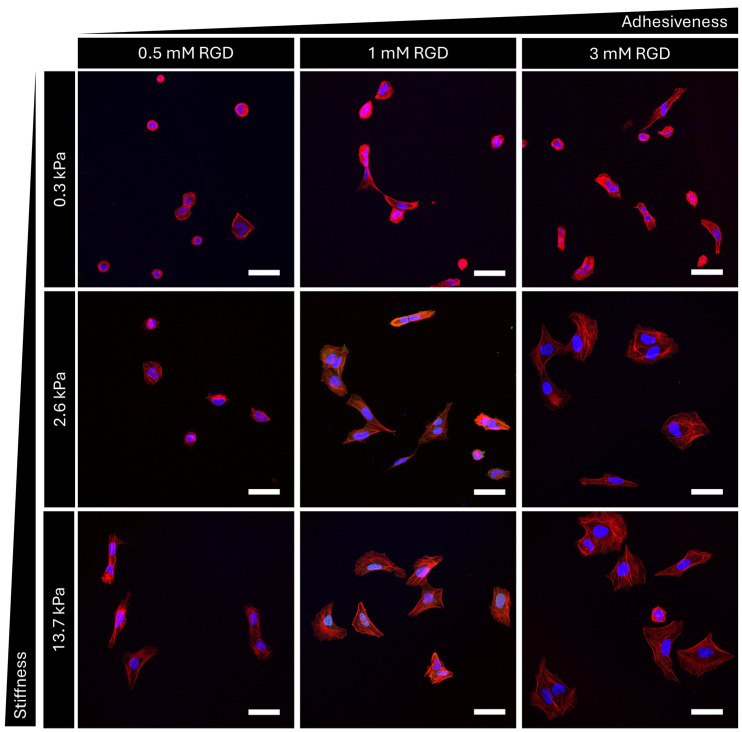
Example of immunofluorescence results performed on U2OS cells seeded on PEG-hydrogel with controlled stiffness and adhesiveness. Nuclei are stained in blue, F-actin in red. Scale bar = 50 μm.

**Figure 8:**
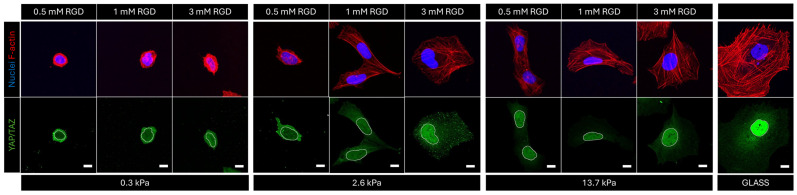
Example of immunofluorescence performed on U2OS cells seeded on PEG hydrogels with controlled stiffness and adhesiveness on a glass substrate (last images on the right). Nuclei are stained in blue, F-actin in red, and YAP/TAZ in green. Scale bar = 10 μm.

Stiffness-tunable hydrogels are crucial tools in biological experiments investigating cell–extracellular matrix interplay. In particular, in the last decades, hydrogels ranging in physiological stiffnesses have been instrumental for mechanobiology studies to unveil the molecular mechanisms determining cellular behavior in healthy and tumor cells. Here we show an example of the use of PEG hydrogels to study how stiffness and adhesiveness play distinct and interdependent roles in mechanosignaling using YAP/TAZ proteins as the readout. To validate the protocol, we synthetized three different hydrogel compositions (namely PEG1, PEG4, and PEG6), each of them with three different concentrations of integrin binding moiety (0.5 mM, 1 mM and 3 mM of RGD peptide). We seeded U2OS cells on the nine different gels, and we performed an immunofluorescence for YAP/TAZ and F-actin, counterstained with a DNA-binding dye (Hoechst). Figure 7 shows a representative field of view for each gel. F-actin is stained in red and the nuclei in blue. As can be seen when comparing the nine images, cells respond to different levels of stiffness and adhesiveness, modifying their shape and internal architecture. Indeed, cell spreading (cell area) and F-actin cytoskeleton organization increase with increasing elastic modulus and RGD concentration. YAP/TAZ localization was then investigated, and results can be seen in Figure 8. As can be seen from cytosolic localization of YAP/TAZ in soft hydrogels, a minimal threshold of stiffness is required for cellular mechanical activation. Then, at medium and high stiffness, adhesiveness can tune the mechanosensing of cells, inducing a shuttling of YAP/TAZ into the nucleus at higher RGD concentration. A complete report of those and additional results can be found in our previous publication [22] where PEG hydrogels were used also to elucidate the role of the nucleus in the cell’s perception of the mechanical characteristics of its microenvironment.

Just to show a comparison, in Figure 8 reports the immunofluorescence analysis for the same U2OS cell seeded on a common culture glass substrate, in which a higher spreading, dense cytoskeleton, and higher level of nuclear YAP/TAZ are evident if compared with cells on the stiffest gels of the protocol.

### 4.3. Troubleshooting

**Figure 9:**
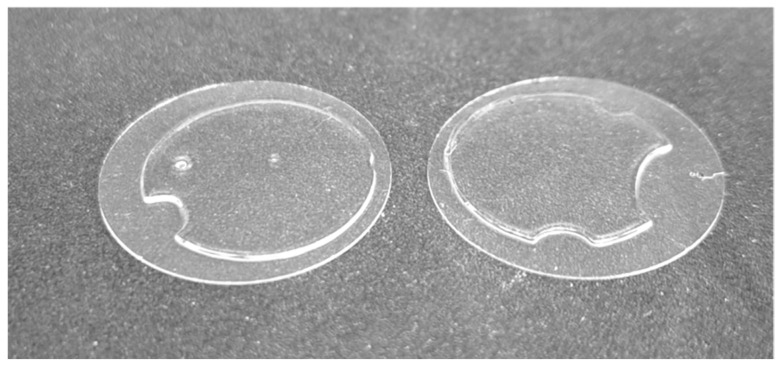
Image of gels resulting from improper deposition of solution.

- In step 33, hydrogels may become difficult to detach from non-adhesive surfaces. To avoid tearing the gel, gently detach the hydrogel with sharp tweezers as soon as the polymerization is completed. Keeping the gels attached for longer periods can lead to partial dehydration
- In step 31, an excessive amount of solution can hinder the correct positioning of the adhesive coverslips on top of the PDMS ring. This can lead to a tilted surface on the gel. Check the proper volume to perfectly fill the mold and press the adhesive coverslip on the PDMS surface to assure proper contact between the two surfaces.
- In step 31, bubbles can occur if the deposition of the adhesive coverslip is performed incorrectly or if the volume of the solution is smaller than necessary. An exemplification is reported in Figure 9. To avoid bubble formation, the glass coverslip should be put in place with a tweezer, starting from one side of the gasket and lowering it gently until the surface completely covers the upper surface of the PDMS. To avoid withdrawing an inaccurate volume due to the viscosity of the solution, low retention tips should be used, and the action should be performed slowly.

- In step 34, be sure that the surface of the polymerized hydrogel is wet. Right after the detachment, before the complete hydration of the surface, gels can show a slightly hydrophobic surface. Use a tweezer to block them and slowly add a drop on top of them till the entire surface is wet.
- In step 34, be sure that all hydrogels are separated from each other before letting them reach swelling equilibrium. If the gel is partially covered during overnight swelling, it may not reach the correct equilibrium.
- In step 37, erroneous sterilization of the glass bottom can lead to hydrogel dehydration. It is mandatory to avoid any contact of the ethanol solution with any part of the hydrogel. The sterilization of the glass can be carried out by touching just the center of the substrate with the sponge and letting the solution spread when the glass is placed inside the multiwell.

## 5. Reagents Setup

Prepare the following solutions before starting the protocol:

1. 3 M NaOH

**Reagent****Final Concentration****Amount**NaOH3 M12 gDI WaterN.A.100 mLTotal
100 mL

2. TMSPM Sol (1% *V*/*V*)

**Reagent**

**Final Concentration**

**Amount**
Ethanol
930 µLAcetic acid5%50 µLTrimethoxysilylpropyl methacrylate (TMSPM)2% *V/V*20 µLTotaln/a1 mL

3. Ethanol 70% Sol (70% *V*/*V*)

**Reagent**

**Final Concentration**

**Amount**
Ethanol70%35 mLWater
15 mLTotal
50 mL

## 6. Conclusions

This protocol outlines a scalable method to produce 2D hydrogel substrates with independent modulation of rigidity and adhesiveness within the range typical of natural tissues. The process requires minimal setup and can be easily implemented in any biological laboratory to study the role of stiffness and adhesivity in cell mechanobiology [2,13,17,24].

Compared to other methods in the literature for synthesizing substrates for similar applications, our protocol offers precise control over both mechanical and cell adhesive properties, utilizing click-reaction polymerization rather than the more commonly used radical polymerization [16,17,25,26,27].

Additionally, this method enables the preparation of adhesive substrates with a broader range of elastic moduli, thanks to the optimization of the synthesis procedure, which surpasses other reported systems [28,29]. The impact of biophysical and adhesive cues on cell behavior has been demonstrated through immunofluorescence analysis, showing modulation of the activation state of the mechanoeffector proteins YAP/TAZ and cell spreading in response to modulations of both stiffness and adhesiveness.

## Figures and Tables

**Table 1:** Procedure steps and time required.

Procedure Step	Time Required
Dimethyl silane functionalization (non-adhesive)	230 min
Methacrylate silane functionalization (adhesive)	90 min
Polymerization set-up preparation	30 min
Hydrogel fabrication	30 min
Hydrogel sterilization (10 gels)	20 min

**Table 2:** PEG hydrogel compositions and stiffness measurements obtained by micropipette aspiration [23].

Hydrogel Composition	8 Arm PEG Final Concentration (wt%)	PBS 1X (μL)	8 Arm PEG-NB Stock (μL)	Adhesive Peptide Stock (μL)	Crosslinking Peptide (μL)	LAP Stock (μL)	Elastic Modulus (kPa)
PEG1	4.7	66.1	18.8	5.3	8.3	1.5	0.30 ± 0.13
PEG2	5.0	63	20	5.3	10.1	1.6	0.87 ± 0.13
PEG3	5.2	60	20.8	5.3	12.3	1.7	1.17 ± 0.31
PEG4	5.5	55.1	21.9	5.3	15.9	1.7	2.63 ± 0.38
PEG5	9	24.5	36	5.3	31.3	2.9	7.71 ± 0.38
PEG6	12.5	1.9	50	5.3	38.9	3.9	13.7 ± 0.48

**Table 3:** UV light intensity and exposure time for hydrogel synthesis.

Hydrogel Composition	UV Light Intensity (mW/cm^2^)	Exposure Time (min)
PEG1	65	10
PEG2	65	10
PEG3	65	10
PEG4	65	10
PEG5	26	2
PEG6	26	2

**Table 4:** Estimated time for the synthesis of 10, 30, or 50 hydrogels.

Hydrogel Composition	Time Required for 10 Gels	Time Required for 30 Gels	Time Required for 50 Gels
Dimethyl silane functionalization (non-adhesive)	230 min	230 min	230 min
Methacrylate silane functionalization (adhesive)	90 min	120 min	140 min
Polymerization set-up preparation	30 min	30 min	30 min
Hydrogel fabrication	30 min	90 min	150 min
Hydrogel sterilization	20 min	25 min	30 min

## Data Availability

Dataset available on request from the authors.
